# Development of a new patient-reported outcome measure for complex cryptoglandular fistulas (20-Item complex cryptoglandular fistula questionnaire^™^): a qualitative study

**DOI:** 10.1186/s41687-024-00729-5

**Published:** 2024-08-22

**Authors:** Jeffrey D. McCurdy, Patrick Crooks, Chad Gwaltney, Robert Krupnick, Kathy-Ann Cadogan, Chitra Karki

**Affiliations:** 1 Department of Medicine, University of Ottawa and Ottawa Hospital Research Institute, General Campus, 501 Smyth Road, Box 206, Ottawa, ON K1H 8L6 Canada; 2 IQVIA Real World Solutions, Patient Centered Solutions, New York, NY USA; 3 Gwaltney Consulting, Westerly, RI USA; 4 IQVIA Real World Solutions, Patient Centered Solutions, Cambridge, MA USA; 5 Takeda Pharmaceuticals USA Inc, Cambridge, MA USA

**Keywords:** Complex cryptoglandular fistula, Patient-reported outcome measure, Symptoms, Impacts

## Abstract

**Background:**

There are limited tools to measure the burden of disease and effectiveness of medical/surgical interventions in patients with cryptoglandular fistulas. The aim of this study was to explore concepts that are relevant and important to patients with complex cryptoglandular fistulas (CCF) and to develop a patient-centred, disease-specific, patient-reported outcome measure (PROM) to assess symptom burden and impacts of CCF.

**Methods:**

A targeted literature review was conducted, followed by one-to-one telephone interviews with five colorectal surgeons (USA, *n* = 3; UK, *n* = 1; Spain, *n* = 1) and 20 US adult patients with CCF to inform the development of a conceptual model and a CCF-specific PROM. The targeted literature review informed the development of the preliminary conceptual model and identified a PROM in the literature that was used as a reference to generate the draft CCF-specific PROM. The colorectal surgeon interviews provided insights on the experience of patients with CCF to refine the conceptual model, formulate probing questions for use in patient interviews, and to develop the draft CCF-specific PROM. Patients’ descriptions of their experiences with symptoms and the impacts on their lives and evaluation of the draft CCF-specific PROM in concept elicitation and cognitive interviews were used to develop the final conceptual model and final CCF-specific PROM.

**Results:**

Ten symptoms (odour, pain during bowel movement, abscess, post-operative pain, discharge/drainage/leakage, anal/perianal pain, inflammation/swelling, skin irritation, bleeding and itchiness) and 11 impacts (discomfort, inability to exercise, embarrassment, difficulty sitting, worry about disease, adapted life to maintain hygiene, negatively impacted social life/isolation, inability to perform daily activities, reduced interest in sex, negatively impacted intimate relationships and negatively impacted mood) were reported as most salient by patients. The patient experience, clinician perspective, and literature review provided input to item generation. Evaluation of relevance and patient understanding through cognitive interviews with patients provided evidence for the content validity of the new patient-reported outcome measure: the 20-item Complex Cryptoglandular Fistula Questionnaire^™^ (CCFQ-20^™^).

**Conclusion:**

The CCFQ-20^™^ is a new clinician-guided, patient-validated, disease-specific patient-reported outcome measure that measures disease impact and quality of life in patients with CCF.

**Supplementary Information:**

The online version contains supplementary material available at 10.1186/s41687-024-00729-5.

## Introduction

Perianal fistulas are chronic abnormal passages, most commonly between the anorectal canal and the perianal skin. Over 90% of perianal fistulas are cryptoglandular in origin [1]. The most widely accepted theory of cryptoglandular fistulas is that they develop from the obstruction and subsequent infection of proctodeal glands, which originate in the intersphincteric plane [1, 2]. Common symptoms include purulent discharge from the external perianal opening and pain [3]. The main form of treatment for cryptoglandular fistulas is surgical drainage and placement of loose fitting setons to prevent recurring infections [4, 5]. The clinical course of cryptoglandular fistulas is highly variable, with many patients requiring recurrent surgical interventions [3].

Cryptoglandular fistulas are broadly defined as ‘simple’ (single, low-lying tract involving less than a third of the sphincter muscles) or ‘complex’ (recurrent, significant involvement of the external sphincter or multiple tracts) [6–8]. Complex cryptoglandular fistulas (CCF) have a substantial impact on quality of life (QoL), causing discomfort and affecting sexual and social functioning [1, 9]. Furthermore, they are associated with substantial healthcare resource utilization [10].

The ultimate goals for the treatment of CCF are to achieve fistula healing and prevent disease recurrence while maintaining continence [6, 7]. Complex fistulas are notoriously difficult to treat, with higher treatment failure rates than simple fistulas and higher risk of recurrence and faecal incontinence [6, 11]. In a recent qualitative study, Iqbal et al. reported the high physical, social and psychological burden borne by patients with cryptoglandular fistulas [12]. To evaluate the burden borne by patients with perianal fistulas, robust and relevant patient reported outcome measures (PROMs) are required. Ferrer-Marquez et al. developed the Quality of Life in Patients with Anal Fistula Questionnaire (QoLAF-Q) for adults with cryptoglandular fistulas; however, this instrument did not involve patients in the item identification and selection, a critical component of PROM development [13]. Further, it was not specific to patients with complex disease, the most clinically relevant disease subset for evaluation in clinical trials. To date, there are no established PROMs to specifically assess the effectiveness of medical and surgical interventions for CCF.

This study had two aims: (1) to explore concepts that are relevant and important to patients with CCF and understand the disease impact on QoL, and (2) to develop a patient-centred, disease-specific PROM to assess the burden of symptoms and their impact on daily life in patients with CCF over time in clinical trials and throughout their disease course in clinical practice.

## Materials and methods

### Study overview

This study used a multi-step iterative process guided by the United States Food and Drug Administration (FDA) and the International Society for Pharmacoeconomics and Outcomes Research (ISPOR) [14–16]. The study was approved by the New England Independent Review Board.

The FDA recommends the development of a conceptual model to define the concepts to be measured by a PROM [14]. A targeted literature search and PROM review were therefore conducted to identify concepts (signs, symptoms and impacts) and existing PROMs relevant to CCF. A preliminary conceptual model was constructed from the targeted literature search and refined after interviews with colorectal surgeons. An initial draft PROM specific to CCF was developed based on the results from the PROM review and evaluated in patient interviews. The preliminary conceptual model and draft PROM were refined and finalized after concept elicitation and cognitive debriefing interviews with patients with CCF to arrive at a final PROM suitable for use in assessing the patient experience.

### Targeted literature search and PROM review

The targeted literature search was conducted using PubMed, Cochrane Library and PsycINFO to identify English language articles published between January 2000 and January 2020 relating to concepts relevant and important to patients with CCF. This timeframe was deemed suitable to identify relevant signs, symptoms and impacts relating to present day management of CCF. Articles were eliminated if they were not related to patient experience, not descriptive of signs/symptoms/potential impacts of disease, focused on pediatric indications or non-human studies or older than 20 years. This informed the development of a preliminary conceptual model. Owing to anticipated limited literature on CCF specifically, other anal fistula types were included in the search.

PubMed, Cochrane Library, ClinicalTrials.gov, the Patient-Reported Outcome and Quality of Life Instruments Database (PROQOLID) and PROLABELS were searched for PROMs/PROM label claims relevant to CCF and related conditions. An updated PROM review was performed in January 2022 to identify any CCF-specific PROMs published between 2020 and 2022. Search terms for the targeted literature search and PROM review are reported in Supplementary Tables S1–S4.

#### Colorectal surgeon interviews

Telephone interviews lasting 60 min each were conducted with five colorectal surgeons from the USA (*n* = 3), UK (*n* = 1) and Spain (*n* = 1). These countries were chosen as representative of a range of healthcare systems across key CCF markets. The number of colorectal surgeons interviewed in this study is consistent with the number of clinical experts interviewed in similar studies for other diseases [17–19]. The aims of these interviews were to explore the surgeons’ perspectives on disease-related signs and symptoms, treatment-related effects and overall impact on patients’ daily lives, to refine the preliminary conceptual model, formulate probing questions for use in patient interviews, and to generate a draft PROM for CCF. Colorectal surgeons with a minimum of 3 years of experience treating patients with CCF and currently managing a minimum of five patients with CCF were recruited via a third-party vendor specializing in recruitment of clinicians for research interviews. Surgeons were chosen because surgery is the main treatment for CCF [20].

### PROM development

Suitable PROM(s) identified in the PROM review/targeted literature search were cross-mapped with concepts identified in the targeted literature search and colorectal surgeon interviews, then used as a reference to develop a draft CCF-specific PROM. This draft PROM was evaluated in the cognitive debriefing part of the patient interviews.

### Patient interviews

Four waves of successive interviews (one-to-one telephone, web-assisted interviews lasting approximately 90 min) each with a different cohort of five US patients with CCF were conducted between May and August in 2020. Patients were recruited by a third-party vendor through social media or physician/site referrals. Inclusion and exclusion criteria are described in Table 1. Interviews were conducted until concept saturation was reached, defined as the point at which no new concepts emerged.

**Table 1:** Eligibility criteria for patient interviews

Inclusion criteria	Exclusion criteria
• Signed informed consent	• Unconfirmed diagnosis of CCF
• 18–75 years old	• Known history of IBD
• Physician-confirmed diagnosis of CCF ≥ 3 months ago^a^	• Unable to provide informed consent
• CCF defined as mid- or high-transsphincteric, horseshoe or branching fistula with either > 1 internal opening or > 1 external opening	
• No history of inflammatory bowel disease (IBD, i.e. ulcerative colitis or Crohn’s disease) or suspected luminal disease	
• Ability to communicate independently on the phone for 90 min	
• Access to the internet during the interview	
• Resident in the USA	

^a^Patients were eligible for inclusion if they had either a physician-confirmed diagnosis of CCF or a simple cryptoglandular fistula with post-surgical recurrence (e.g., a failed fistulotomy, advancement flap or ligation of the intersphincteric fistula tract procedure) or were ineligible for a fistulotomy, provided they met all other inclusion criteria. *CCF* complex cryptoglandular fistula, *IBD* inflammatory bowel disease

Each semi-structured interview consisted of two parts, concept elicitation and cognitive debriefing. Four interviewers trained in interviewing patients for concept elicitation and cognitive debriefing took part in the study. Interviews were based on a patient discussion guide consisting of open-ended questions with probes, developed from the targeted literature search and colorectal surgeon interviews.

#### Concept elicitation

Patients were asked about their experience of living with CCF in order to develop a final conceptual model. The concept elicitation explored words and phrases patients used to describe CCF, timing and triggers of symptoms and symptom evolution, symptom characteristics (elicited using both open-ended and probing questions) and association with treatment, the impacts of symptoms on the patient’s life, and the degree of disturbance on the patient’s life owing to symptoms and impacts. For each symptom and impact, patients rated the disturbance of the concept on a 10-point numerical scale (0 = ‘not disturbing at all’ to 10 = ‘extremely disturbing’). Symptoms and impacts that were mentioned by ≥ 50% of patients and that had an average disturbance rating ≥ 5 were considered most salient. Concept saturation was assessed during concept elicitation.

#### Cognitive debriefing and PROM refinement

In cognitive debriefing, the draft CCF PROM was evaluated. Patients provided feedback on the instructions, items and response options of the draft CCF PROM. An item generation workshop was conducted to review feedback received from each wave of interviews and determine changes to be made to the items for the next wave of interviews. This process was repeated for each of the four waves of interviews, as needed. In addition, the patient’s thought process as they completed the draft CCF PROM was evaluated, and any aspects of the PROM that were confusing, unclear or problematic, or prompted suggestions for improvements, were identified.

### Analysis

A thematic coding approach was used to code key signs, symptoms and impacts of CCF. ATLAS.ti v8 software was used to code the de-identified transcripts of the patient interview recordings. Predetermined coding rules were used to ensure accuracy and consistency of coding. Two trained coders completed the first three transcribed interviews until an inter-coder agreement (ICA) of *r* ≥ 0.7 was achieved (*r* = 1.0: complete agreement). ICA was calculated using the ATLAS.ti v8 software. After the ICA threshold was achieved, approximately every fifth transcript was double-coded to ensure consistency of coding. Coding was completed in the order that the interviews were conducted. For all concepts, the number of patients who reported the concept, average disturbance ratings and concept saturation analyses were calculated.

## Results

### Targeted literature search and PROM review

**Fig. 1:**
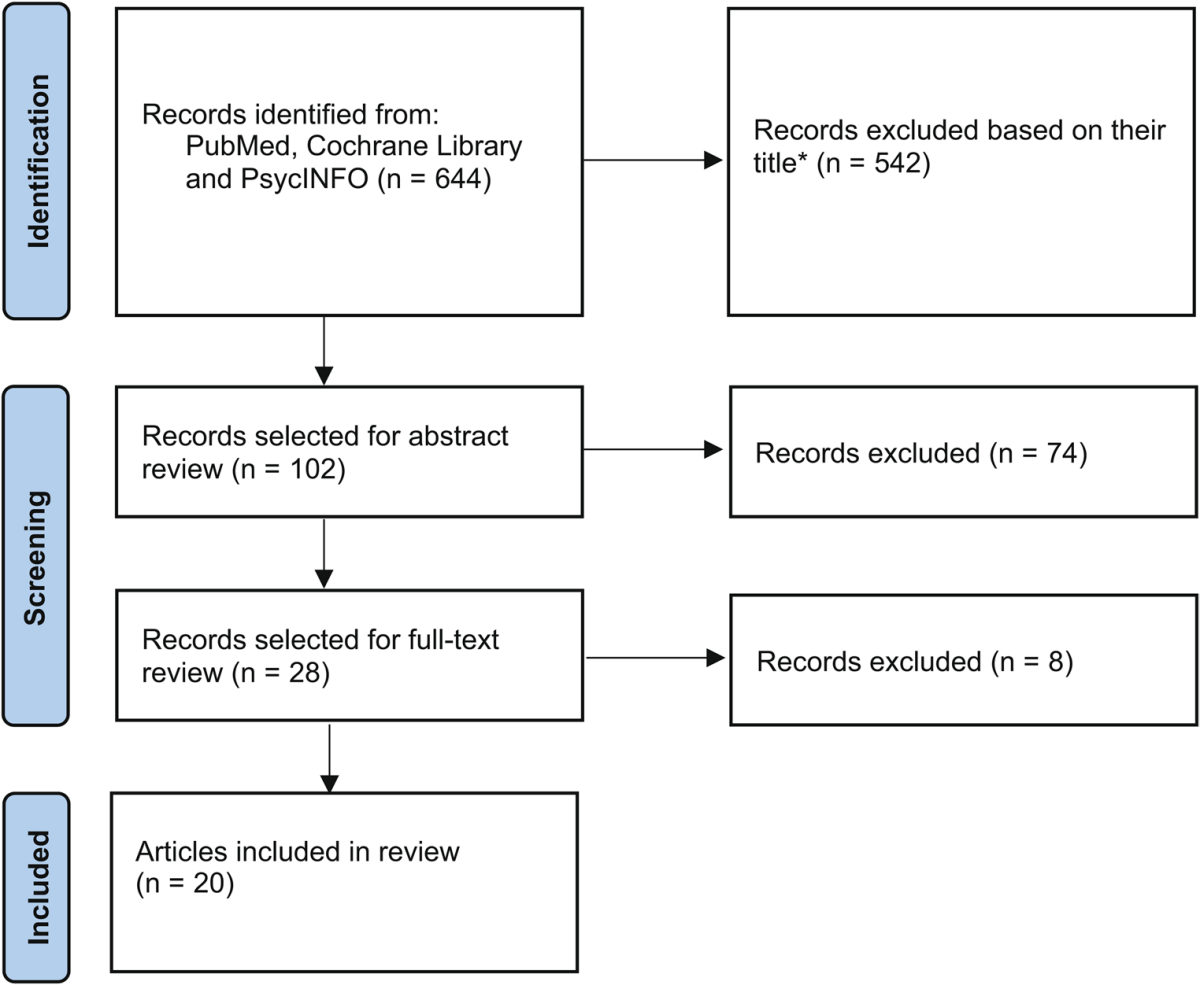
Flow chart outlining article selection process. *Articles were eliminated if they were not related to patient experience, not descriptive of signs/symptoms/potential impacts of disease, focused on pediatric indications or non-human studies or older than 20 years

The targeted literature search yielded 644 articles of which 20 were identified as relevant (Fig. 1 and Supplementary Table S5) and used to develop the preliminary conceptual model (Supplementary Figure S1) [13, 21–39]. Only 3 articles focused on CCF [24, 25, 39]. Discharge, pain, faecal incontinence and bleeding were the most prevalent symptoms across anal fistula types and were also reported in articles that focused on CCF. Embarrassment was the most prevalent reported impact of CCF. Minimal qualitative patient data were available from the identified articles, and the concepts in the preliminary conceptual model were mainly based on assessment scales, questionnaires and state-of-the-science reviews. As a result, impacts extracted from these data sources were described using broad terminology.

The targeted literature search and PROQOLID/PROLABELS searches identified two and four PROMs, respectively. The QoLAF-Q was the only PROM identified that was specific to cryptoglandular anal fistulas [13]. This PROM consists of 14 items (seven items on physical impact of anal fistulas and seven items on biopsychosocial impact of anal fistulas) measured on a 5-point Likert-type scale, and it was psychometrically evaluated in patients with cryptoglandular fistulas with no history of inflammatory bowel disease [13].

### Colorectal surgeon interviews

Colorectal surgeons most frequently cited discharge, perianal pain, abscess and infection/sepsis as symptoms associated with CCF. The most commonly occurring and bothersome symptoms identified by the surgeons were perianal pain and discharge. The treatment effects (adverse events) most frequently cited included incontinence (gas and/or faecal), post-operative pain and recurring abscess/fistula. The most frequently cited impacts included depression, taking time off school/work and inability to perform daily activities. All new symptoms and impacts reported by the colorectal surgeons were used to refine the preliminary conceptual model.

### PROM development

Based on the concepts identified in the targeted literature search and colorectal surgeon interviews, the QoLAF-Q was observed to include many of the key concepts related to CCF. Nevertheless, prior to patient interviews, the QoLAF-Q was modified by revising the wording of the items to aid patient understanding and revising the response options for simplification to create a draft CCF PROM. This was used in the first wave of patient interviews.

### Patient interviews

#### Patient demographics and disease characteristics

In total, 20 US adults with CCF were interviewed. Demographics and disease characteristics are summarized in Table 2. Mean patient age was 49 years (standard deviation [SD], 10.0), the majority were female (60%), and the mean time from diagnosis to interview was 21.9 months (SD, 35.4). The dominant anatomic fistula subtype was transsphincteric fistula (55%) and 40% had a horseshoe extension. All patients had undergone drainage as part of their treatment for CCF.

**Table 2:** Patient demographic and disease characteristics^a^ (*N* = 20)

	*N* = 20
Age, years	
Mean (SD)	48.6 (10.0)
Median	48
Range	32–69
Sex, *n* (%)	
Male Female Ethnicity, *n* (%) White Hispanic Black White/Asian	8 (40) 12 (60) . 9 (45) 7 (35) 3 (15) 1 (5)
Diagnosis subtype (some patients had > 1 type), *n* (%)	
Mid- or high-transsphincteric	11 (55)
Horseshoe	8 (40)
Branching fistula with either > 1 internal opening or > 1 external opening	5 (25)
Anterior/intersphincteric	2 (10)
Treatments undergone for CCF (some patients had multiple treatments), *n* (%)	
Seton/drainage	20 (100)
Plug	10 (50)
Fistulotomy	9 (45)
Antibiotics/advancement flap/LIFT/partial fistulectomy/stem cell implants	8 (40)
Time since diagnosis, months^b^	
Mean (SD)	21.9 (35.4)
Range Median	2.5–170.5 11.0

^a^Patients involved in concept elicitation and cognitive debriefing interviews

^b^Time since diagnosis data are approximations because date for diagnosis was reported as month and year and for 1 patient, only year (diagnosed in 2006)

*CCF* complex cryptoglandular fistulas, *LIFT* ligation of the intersphincteric fistula tract, *SD* standard deviation

### Concept elicitation

In total, 26 symptoms (Table 3) and 35 impacts (Table 4) arose from concept elicitation interviews, of which 10 symptoms and 11 impacts were identified as most salient. The most salient symptoms were odour, pain during bowel movement, abscess, post-operative pain, discharge/drainage/leakage, anal/perianal pain, inflammation/swelling, skin irritation, bleeding and itchiness. The most salient impacts were discomfort, inability to exercise, embarrassment, difficulty sitting, worry about disease, adapted life to maintain hygiene, negatively impacted social life/isolation, inability to perform daily activities, reduced interest in sex, negatively impacted intimate relationships and negatively impacted mood. The topmost salient (frequently reported with the highest disturbance rating) symptoms and impacts were odour, pain on bowel movement, abscess, discomfort, embarrassment, worry about disease, negatively impacted social life/isolation, adapted life to maintain hygiene and negatively impacted intimate relationships.

**Table 3:** Symptoms reported by patients during patient interviews

Symptom	Patients reporting symptom^a^ *N* = 20	Disturbance rating, mean (*n*)^b^	Patients reporting spontaneously *N* = 20	Patients reporting after probing *N* = 20
Discharge/drainage/leakage^c^	19	7.3 (15)	11	8
Bleeding^c^	18	6.9 (14)	15	3
Abscess^c^	18	7.7 (14)	12	6
Skin irritation (soreness, redness, tenderness)^c^	17	6.9 (15)	10	7
Inflammation/swelling^c^	17	7.0 (15)	8	9
Odour^c^	16	9.1 (16)	14	2
Pain when having bowel movement^c^	16	8.6 (15)	13	3
Anal/perianal pain^c^	15	7.2 (13)	14	1
Itchiness^c^	15	6.1 (15)	4	11
Post-operative pain^c^	11	7.4 (7)	4	7
Fibrosis/scarring	10	4.7 (7)	1	9
Constant feeling of needing to defecate	7	7.3 (7)	0	7
Infection symptoms (feeling feverish, sick, low energy)	6	8.3 (4)	6	0
Constipation	5	8.3 (4)	5	0
Recurring abscess/fistula	5	6.0 (1)	5	0
Incontinence (gas)	5	6.4 (5)	1	4
Incontinence (faecal)	4	8.3 (3)	2	2
Wound discharge/drainage	4	3.8 (4)	1	3
Ulceration	4	7.7 (3)	0	4
Diarrhoea	3	9.3 (3)	3	0
Tummy cramps/pain	2	4.0 (1)	2	0
Dehiscence of wound/burst stitches	1	10.0 (1)	1	0
Puckering	1	10.0 (1)	1	0
Muscle spasms	1	8.0 (1)	1	0
Internal burning	1	8.0 (1)	1	0
Keyhole deformity/denting	1	0.0 (1)	0	1

^a^Either spontaneously or after probing in patient interviews

^b^When ‘n’ in disturbance ratings deviates from ‘n’ in patients reporting symptoms, this indicates missing data

^c^Symptoms identified as being most salient, defined as mentioned by ≥ 50% of patients and having a mean disturbance rating ≥ 5 on a 10-point scale (0 = not disturbing at all, 10 = extremely disturbing)

**Table 4:** Impacts reported by patients during patient interviews

Impact	Patients reporting impact^a^ *N* = 20	Disturbance rating, mean (*n*)^b^	Patients reporting spontaneously *N* = 20	Patients reporting after probing *N* = 20
Discomfort/uncomfortable^c^	18	8.4 (9)	15	3
Inability to exercise^c^	17	7.5 (15)	9	8
Adjusted diet	16	4.4 (9)	15	1
Difficulty sitting^c^	16	7.4 (13)	13	3
Embarrassment^c^	16	8.8 (13)	11	5
Worry about disease^c^	15	8.0 (11)	10	5
Increased preparation/adapt life to maintain hygiene (e.g. wear pads)^c^	14	8.3 (3)	13	1
Negatively impacted social life/isolation^c^	14	7.9 (11)	10	4
Inability to perform daily activities^c^	14	6.7 (11)	8	6
Reduced interest in sex^c^	13	6.6 (8)	8	5
Negatively impacted intimate relationships^c^	12	9.1 (10)	6	6
Impacted mood (e.g. depression, sadness)^c^	10	8.0 (8)	7	3
Urgency	9	5.7 (7)	3	6
Burden of treatment/fear of treatment failure	8	8.3 (4)	5	3
Taking time off school/work	7	7.8 (4)	4	3
Inability to maintain hygiene	7	8.0 (4)	2	5
Soiling	7	7.0 (3)	2	5
Sleep disturbance	7	6.5 (6)	1	6
Low self-esteem	6	10.0 (1)	5	1
Permanent scarring	4	10.0 (1)	4	0
Spending lots of time in bathroom	4	5.0 (2)	4	0
Stress on family	4	7.7 (3)	0	4
Fear of going to the bathroom	3	10.0 (1)	3	0
Difficulty laying on back	3	7.0 (1)	3	0
Limited diet	3	10.0 (1)	2	1
Financial impact	3	10.0 (1)	1	2
Inability to have sex	3	8.3 (3)	0	3
Feeling withdrawn	2	10.0 (1)	2	0
Frustration	2	10.0 (1)	2	0
Avoidance of eating^d^	2	N/A	2	0
Weight gain^d^	2	N/A	2	0
Anger	1	10.0 (1)	1	0
Anxiety^d^	1	N/A	1	0
Avoidance of going to bathroom^d^	1	N/A	1	0
Prevented from having further children^d^	1	N/A	1	0

^a^Either spontaneously or after probing in patient interviews

^b^When ‘*n*’ in disturbance ratings deviates from ‘*n*’ in patients reporting symptoms, this indicates missing data

^c^Impacts identified as being most salient, defined as mentioned by ≥ 50% of patients and having a mean disturbance rating ≥ 5 on a 10-point scale (0 = not disturbing at all, 10 = extremely disturbing)

^d^Avoidance of eating, weight gain, anxiety, avoidance of going to bathroom, and prevented from having further children were mentioned by patients, but no rating provided

*N/A* not applicable

Patients used strong terminology and language to describe the odour they experienced. One patient noted:

It was a stinky, foul odour, like something died. (Patient 1, male, 47 years old).

Patients described the pain experienced during bowel movements as a ‘burning sensation’, ‘being irritated when pooping’, a ‘dull pain’, a ‘pressure’, ‘sensitive’, ‘discomfort’, and ‘soreness’, noting:

Going to the bathroom it was painful. (Patient 18, male, 44 years old).

The pressure build-up from the formation of an abscess resulted in pain for several patients at times other than during a bowel movement. In some instances, patients drew on the example of haemorrhoids or an infected pimple to describe their experience of this pain, noting:

The pain of a haemorrhoid, if you’ve ever experienced that, is something that’s very closely related to the pain I felt with this. (Patient 2, female, 57 years old).

Patients described a general feeling of discomfort throughout their experience with CCF. This was caused by several factors such as pain, swelling, and placement of a seton.

You’ve got a drain coming out, so it’s kind of awkward to be sitting, or laying, so that was very uncomfortable, especially the first week. (Patient 12, female, 41 years old).

Feeling embarrassed was linked most strongly with the experience of an unpleasant odour or gas incontinence, and the resulting impact on social situations with friends and/or intimacy with partners. One patient reported:

I would have to say with my wife I felt just embarrassed by the whole thing, just the smell, and I just couldn’t really get intimate with her because it bothered me. (Patient 9, male, 42 years old).

Patients also experienced periods of worry about their condition, whether a treatment might be successful, and if the fistula would return, noting:

Well, it’s affected where if I don’t keep drinking water and if I don‘t keep up with all the fiber, then I’m worried to get that fistula again, that blockage. So I guess it is still affecting my life. (Patient 14, female, 51 years old).

Patients described the negative impact of CCF on their social life and limiting their social activities owing to being self-conscious about their condition, in particular owing to experiencing an unpleasant odour or needing the bathroom frequently.

…from the time it was going on until the time that it was remedied, I curtailed my social life quite a bit to the point where I wasn’t associating with friends, going out and doing the things that I felt comfortable doing…You just feel like basically withdrawing. (Patient 8, male, 54 years old).

Patients also introduced additional steps into their daily routine in order to manage specific symptoms, such as drainage and odour. These included wearing sanitary pads, going often to the bathroom, and increased frequency of washing.

Well, I have pads with me all the time. Lots of time spent in the bathroom. Lots of baby wipes. Initially, when I was having all the surgeries, I lived with a sitz bath. A sitz bath, a little enema thing, and you’re just cleaning, cleaning, cleaning. (Patient 5; female, 49 years old).

Many patients described not being interested in sexual activity with their partner, reduced intimacy overall, and avoidance of dating owing to their CCF.

“If I get asked out on a date, I don’t even feel like meeting anybody to even get to the point of getting intimate because I don’t even want to go… You know what I’m saying? (Patient 7, female, 43 years old).

Supplementary Tables S6–S14 include more illustrative quotes from patients and Supplementary Figures S2 and S3 show frequency and disturbance ratings for the most salient symptoms and impacts. Concept saturation for symptoms was reached after the second wave of interviews and after the third wave for impacts.

#### Cognitive debriefing and PROM refinement

During wave 1 cognitive debriefing interviews, patients reported that the key concepts were covered by the draft CCF PROM (pain, discharge incontinence, mental and physical health, sexual impact); however, some concepts were missing. Additionally, the lack of a specific timeframe/recall period created confusion for the patients given their symptom fluctuation and evolution over time. The following concepts were added to the draft CCF PROM based on the concept elicitation part of wave 1 interviews: odour, pain during bowel movements, skin irritation, the presence of an abscess, impact on work/school and impact on daily activities. Two concepts were removed from the draft CCF PROM (other aspects of life and level of independence) to focus the questionnaire on CCF-specific concepts and impact on daily activities. A 7-day recall period was included in the draft CCF PROM to enable patients to accurately recall their symptoms and for tracking changes over time. The response options were adjusted from 4 to 5 to provide more granularity in symptoms and impact reporting and to align with the QoLAF-Q PROM.

The draft CCF PROM was further revised after the wave 2 interviews. A question on overall health was removed to focus the PROM on CCF-specific concepts only. Modifications were made to some items and wording to improve relevance (e.g., for items assessing skin irritation, ‘itchiness’ was included in the description to be more inclusive of patients’ experience of skin irritation) and patient understanding (e.g., the description of fistula discharge was adjusted to clarify that any leaked faecal matter was most likely liquid).

Overall, the PROM items were very well-received at wave 3 interviews. They were considered relevant by patients and no key concepts were perceived as missing. Modifications at this stage were for clarity; no new concepts were added or removed. Overall, the items were well received at wave 4, described as easy to understand and answer. After wave 4 interviews, the PROM was finalized by making adjustments to the wording of several items to increase clarity and descriptions of two concepts to optimize relevance.

#### Final conceptual model and final CCF PROM

**Fig. 2:**
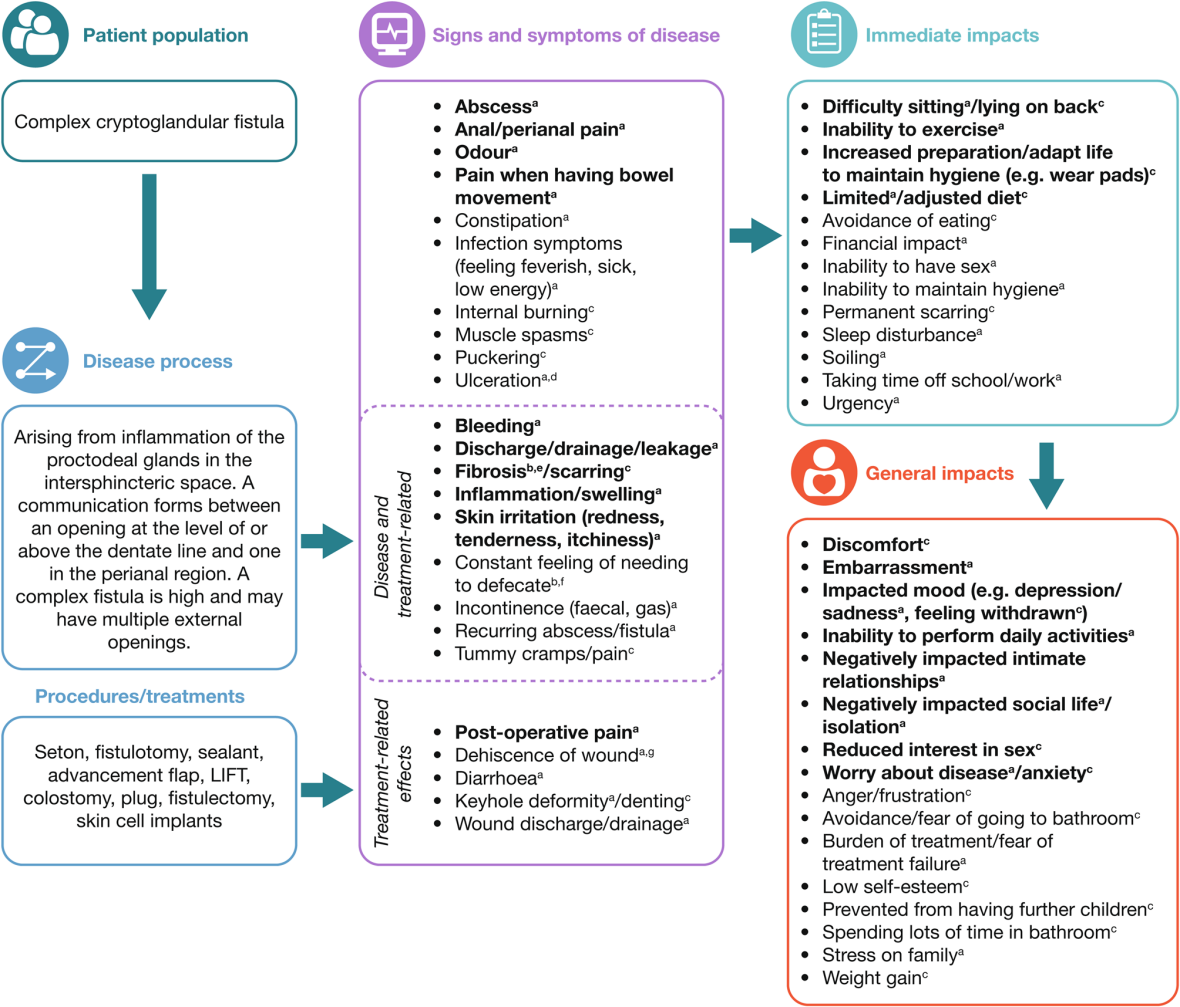
Final CCF conceptual model. CCFQ-20™ is copyright of Takeda Pharmaceuticals 2022. Bold indicates concepts experienced by ≥ 10 patients. ^a^Concepts derived from the literative review. ^b^Concepts added after the clinician interviews. ^c^Concepts added after the patient interviews. ^d^Formation of ulcers. ^e^Thickening and scarring of connective tissue. ^f^Tenesmus: a continual or recurrent inclination to evacuate the bowels. ^g^Separation of wound. Abbreviations: CCF, complex cryptoglandular fistulas; CCFQ-20™; 20-item Complex Cryptoglandular Fistula Questionnaire™; LIFT, ligation of the intersphincteric fistula tract

All new concepts reported by patients in the concept elicitation interviews were added to the post-clinician interview conceptual model to produce the final conceptual model (Fig. 2). The final CCF PROM, the 20-item Complex Cryptoglandular Fistula Questionnaire^*™*^ (CCFQ-20^™^), consisted of six symptom categories (discharge, incontinence, pain, irritation, odour and abscess) and four QoL categories (functional, physical health, psychological health and social health) (Table 5). It had a total of 20 items, of which 14 were related to symptoms of CCF and six to impacts on QoL. It included a 7-day recall period and a Likert-type scale with five response options (except for items assessing abscess presence and impact on work/school, where yes and no answers were used or quantification [hours] were requested). Overall, the final CCFQ-20^™^ was relevant to patients’ experience with CCF.

**Table 5:** Categories and measures in the final 20-item Complex Cryptoglandular Fistula Questionnaire^™^ (CCFQ-20^™^)

Categories	Measures
Discharge, incontinence and odour	Frequency, amount/intensity
Pain and irritation	Frequency and severity/intensity
Abscess	Presence
Quality of life: • Functional • Physical health • Psychological health • Social health	Impact

CCFQ-20™ is copyright of Takeda Pharmaceuticals 2022

## Discussion

The CCFQ-20^™^ is a new patient-centred, disease-specific PROM developed to measure disease burden and QoL in patients with CCF. It was developed using the QoLAF-Q as a base and through a multi-step, iterative process involving a review of published literature, consultation with colorectal surgeons, and interviews with patients to identify 10 key symptoms and 11 key QoL impacts associated with CCF.

A recently published systematic review by Iqbal et al. assessed the content validity of instruments used to assess QoL in patients with cryptoglandular fistulas using the COnsensus-based Standards for the selection of health Measurement Instruments (COSMIN) methodology [40]. This study concluded that the development of such instruments (including the Qo-LAF-Q) lacked construct definition, methodological detail, patient involvement in development, and cognitive interviews or pilot testing, and did not meet the required quality according to COSMIN standards [40]. We based the CCFQ-20^™^ on the QoLAF-Q because it was the only PROM specific to cryptoglandular anal fistulas identified at the time of development. However, unlike the QoLAF-Q, the CCFQ-20 was developed in line with FDA regulatory guidance and included in-depth qualitative interviews with patients to identify items relevant to them [14]. Six new concepts were added to the CCFQ-20^™^ (odour, pain during bowel movements, skin irritation, the presence of an abscess, impact on work/school and impact on daily activities), three concepts were removed (overall health, level of independence, and other aspects of life) and a 7-day recall period was introduced. Furthermore, our PROM contains elements of the core outcomes identified by Iqbal et al. that they considered should be measured in studies evaluating fistula treatment, such as fistula symptoms, incontinence, complications and QoL [41]. The patient-centred approach ensured that the CCFQ-20^™^ included the concepts that were most frequently experienced and had a high impact on patients’ lives, thus ensuring content validity. Concept saturation was achieved for both symptoms and impacts, indicating that the content of the PROM was valid. Changes made throughout the cognitive debriefing interviews ensured that the CCFQ-20^™^ was relevant and easy to understand with appropriate response options. The 7-day recall period of the CCFQ-20^™^ is in line with FDA guidelines which state that short recall periods are preferable to long recall periods [14].

The majority of the most salient symptoms and impacts reported by patients in this study are consistent with the findings of a recent qualitative investigation of patients’ experience with cryptoglandular fistulas carried out by Iqbal et al. [12]. In both that study and ours, patients reported symptoms of pain, discharge, systemic symptoms and abscess, and impacts on their daily life and activities, work/school, relationships/intimacy and psychological health. A previous study by Wong et al. has shown differences between what surgeons and patients consider to be important QoL factors associated with anal fistulas and their treatment [42]. This indicates a need for a patient-centred PROM that assesses factors that are specifically important to patients. Concepts reported by patients in the Wong et al. study and ours are pain, continence, psychological health, leakage, work, social activity, sexual function and bleeding [42].

To our knowledge, the CCFQ-20^™^ is the first and only patient-centric PROM designed exclusively for, and based on the experiences of, patients with CCF. The primary goal of this PROM is for use in clinical trials to evaluate the benefits of curative and symptom-reducing interventions over time. Prior to this, psychometric analyses are required to confirm that the measurement properties (test-retest reliability, internal consistency, construct validity, and sensitivity to change) of the CCFQ-20 are fit for use in clinical trials. Linguistic and cultural validation will also be required. The heterogeneity in definitions used to measure the success of interventions in CCF presents challenges when synthesizing the available data and comparing the relative effectiveness of different interventions [43]. It is hoped that the CCFQ-20^™^ may help to standardize research, improve study quality, and allow for more accurate data synthesis in clinical trials. Furthermore, it has the potential to be used in clinical practice to monitor patients’ fistula experience over time and guide decision-making.

The CCFQ-20^™^ PROM has several strengths. Interviews were conducted until concept saturation was reached, indicating the sample size was sufficient. Owing to the patient-centred methodological approach, it is highly relevant to patients’ experience with CCF, as well as being clear and easy to understand. The CCFQ-20^™^ has also been informed by concepts identified in the literature and input from expert colorectal surgeons.

There are several limitations that should be acknowledged. Only 3 of the articles in the literature review focused on CCF. However, all the articles except one (which focused on Crohn’s disease) focused on anal fistulas. The symptoms and impacts in the 3 articles were not dissimilar to the other articles used to inform the model. Furthermore, given that 90% of anal fistulas are cryptoglandular in origin, on balance we felt it was more informative to use a wider range of literature, rather than draw from only three articles. Additionally, the symptoms and impacts of perianal fistulas on patient QoL are similar regardless of whether the cause is cryptoglandular in origin or Crohn’s disease [44, 45]. The patient sample may not be representative of the wider population of patients with CCF, given the lack of heterogeneity in the treatment procedures (more fistulotomies than advancement flaps or ligation of the intersphincteric fistula tract procedures). The study population had a high rate of treatment success, with only a small number of patients experiencing fistula reoccurrence and/or treatment failure. In light of this, some items in the CCFQ-20^™^ that are associated with treatment failures and/or fistula reoccurrence (e.g., faecal incontinence) were retained irrespective of whether they were reported as highly salient by patients.

The patient sample may also not be representative of the population of patients with CCF in terms of ethnic diversity. While data on ethnicity among patients with CCF in the USA is lacking, the patient sample was not representative of the general US population. In this study, there was a lower proportion of White participants and Asian participants and a higher proportion of Hispanic participants when compared with the general US population [46]. The lack of ethnic diversity in this study also precluded any evaluation of differences in signs/symptoms across ethnicities. This may be an important inclusion for future model refinements so that it is applicable for all ethnicities. Another potential limitation is the inclusion of only colorectal surgeons in our study and not advanced practice providers (physician assistants and nurse practitioners) who have an extensive role in patient care in anorectal fistula disease. As is the case with all PROM development, patient responses may have been subject to recall bias.

## Conclusion

The CCFQ-20^™^ is the first patient-centric PROM for CCF developed and assessed for content validity, following expert guidance, regulatory best practices, and patient input, and including a comprehensive battery of salient symptoms and impacts appropriate for evaluating the burden of symptoms and their impact on a patient’s daily life. Future psychometric analyses will seek to confirm the external validity of the CCFQ-20^™^ and support its use in clinical trials and observational studies.

## Electronic supplementary material

Below is the link to the electronic supplementary material.

Supplementary Material 1

## Data Availability

The datasets used and/or analyzed during the current study are available from the corresponding author on reasonable request.

## Abbreviations

CCF: Complex cryptoglandular fistulas
CCFQ-20™: 20-item Complex Cryptoglandular Fistula Questionnaire*™*
COSMIN: COnsensus-based Standards for the selection of health Measurement Instruments
FDA: United States Food and Drug Administration
IBD: Inflammatory bowel disease
ICA: Inter-coder agreement
IRB: Independent review board
ISPOR: International Society for Pharmacoeconomics and Outcomes Research
LIFT: Ligation of the intersphincteric fistula tract
PROM: Patient-reported outcome measure
PROQOLID: Patient-Reported Outcome and Quality of Life Instruments Database
QoL: Quality of life
QoLAF-Q: Quality of Life in Patients with Anal Fistula Questionnaire
SD: Standard deviation
