# Supplementary material for: Development of a new patient-reported outcome measure for complex cryptoglandular fistulas (20-Item complex cryptoglandular fistula questionnaire™): a qualitative study
Source: J Patient Rep Outcomes. 2024 Aug 22;8:99. doi: 10.1186/s41687-024-00729-5 (PMC11341802; doi:10.1186/s41687-024-00729-5)
Supplement: Supplementary file 1 — Supplementary Material 1 [file 41687_2024_729_MOESM1_ESM.docx]

# Supporting information

## Table S1 Search terms for CCF concepts (signs, symptoms and impacts) using the PubMed, Cochrane Library and PsycINFO databases

| 1 | A search of targeted disease terminology, across all article text: “complex cryptoglandular fistula” OR “high trans-sphincteric fistula” OR “mid trans-sphincteric fistula” OR “anterior fistula”) OR “horseshoe fistula” OR “branching fistula” |
| --- | --- |
| 2 | A search including broader disease terms but focusing on title/abstract text only: "complex cryptoglandular fistula” OR "CCF“ OR "high trans-sphincteric fistula" OR "mid trans-sphincteric fistula" OR "anterior fistula" OR "horseshoe fistula" OR "branching fistula“ OR "complex fistula" OR "cryptoglandular fistula" OR "anal fistula“ OR "fistula-in-ano" OR "intersphincteric fistula" OR "low trans-sphincteric fistula" OR "extrasphincteric fistula" OR "suprasphincteric fistula" OR "anal abscess" OR "perianal abscess“ OR "anorectal abscess" OR "anal cyst" OR "perianal cyst” OR "anorectal cyst“ |
| 3 | Each search also looked for relevant signs and symptoms terminology within titles/abstract text: AND “sign” OR “symptom” OR “quality of life” OR “QoL” OR “clinical manifestation” OR “disease model” OR “HRQoL” OR “conceptual model” OR “patient perspective” OR “patient centric” OR “patient centered” or “patient reported” OR “health status” OR “depression” OR “anxiety” OR “work productivity” OR “incontinence” OR “continence” OR “health utility” OR “social life” OR “emotional impact” OR “disability” |

Abbreviations: CCF, complex cryptoglandular fistula; QoL, quality of life; HRQoL, health-related quality of life.

## Table S2 Search terms for the PROM review using the ClinicalTrials.gov database

The following search terms were used to search ClinicalTrials.gov for the use of clinical outcome assessments (separate searches were conducted owing to character limits when searching ClinicalTrials.gov).

| 1 | “Complex cryptoglandular fistula OR high trans-sphincteric fistula OR mid trans-sphincteric fistula OR anterior fistula OR horseshoe fistula OR branching fistula” |
| --- | --- |
| 2 | “Complex fistula OR cryptoglandular fistula OR anal fistula OR fistula-in-ano OR intersphincteric fistula OR low trans-sphincteric fistula OR extrasphincteric fistula” |
| 3 | “suprasphincteric fistula OR anal abscess OR perianal abscess OR anorectal abscess OR anal cyst OR perianal cyst OR anorectal cyst” |
| 4 | “Perianal fistula” (added as a search term as per a suggestion of ClinicalTrials.gov, which identified it as a relevant related term) |
| 5 | “Anorectal fistula” (added as a search term as per a suggestion of ClinicalTrials.gov, which identified it as a relevant related term) |

Abbreviation: PROM, patient-reported outcome measure.

## Table S3 Search terms for the PROM review using the PROQOLID and PROLABELS databases

| 1 | “Complex cryptoglandular fistula” OR “CCF” OR “high trans-sphincteric fistula” OR “mid trans-sphincteric fistula” OR “anterior fistula” OR “horseshoe fistula” OR “branching fistula” |
| --- | --- |
| 2 | “Complex fistula” OR “cryptoglandular fistula” OR “anal fistula” OR “fistula-in-ano” OR “intersphincteric fistula” OR “low trans-sphincteric fistula” OR “extrasphincteric fistula” OR “suprasphincteric fistula” OR “anal abscess” OR “perianal abscess” OR “anorectal abscess” OR “anal cyst” OR “perianal cyst” OR “anorectal cyst” |
| 3 | “Patient reported outcome” or “PRO” or “patient reported outcome assessment” OR “quality of life” OR “assessment” OR “instrument” OR “QoL” OR “HRQoL” OR “health status” OR “health utility” |

Abbreviations: CCF, complex cryptoglandular fistula; HRQoL, health-related quality of life; PRO, patient-reported outcome; PROM, patient-reported outcome measure; PROQOLID, Patient-Reported Outcome and Quality of Life Instruments Database; QoL, quality of life.

## Table S4 Search terms for the PROM review using PubMed and Cochrane Library databases

| 1 | “Complex cryptoglandular fistula” OR “CCF” OR “high trans-sphincteric fistula” OR “mid trans-sphincteric fistula” OR “anterior fistula” OR “horseshoe fistula” OR “branching fistula” |
| --- | --- |
| 2 | “Complex fistula” OR “cryptoglandular fistula” OR “anal fistula” OR “fistula-in-ano” OR “intersphincteric fistula” OR “low trans-sphincteric fistula” OR “extrasphincteric fistula” OR “suprasphincteric fistula” OR “anal abscess” OR “perianal abscess” OR “anorectal abscess” OR “anal cyst” OR “perianal cyst” OR “anorectal cyst” |
| 3 | Instruments identified from the ClinicalTrials.gov and PROQOLID searches as well as any additional relevant instruments identified in the concept identification literature review |
| 4 | “Psychometric” OR “MCID” OR “MID” OR “meaningful change” OR “validity” OR “reliability” OR “measurement” |

CCF, complex cryptoglandular fistulas; MCID, minimal clinically important difference; MID, minimal important difference; PROM, patient-reported outcome measure; PROQOLID, Patient-Reported Outcome and Quality of Life Instruments Database.

## Table S5 Articles identified in the targeted literature review

|  | **Title** | **Reference** |
| --- | --- | --- |
| 1 | Anorectal infection: abscess-fistula | Abcarian et al. 2011 |
| 2 | Anal fistula plug: a prospective evaluation of success, continence and quality of life in the treatment of complex fistulae | Adamina et al. 2014 |
| 3 | Symptom amelioration in Crohn's perianal fistulas using video-assisted anal fistula treatment (VAAFT) | Adegbola et al. 2018 |
| 4 | Advancement flap for treatment of complex cryptoglandular anal fistula: prediction of therapy success or failure using anamnestic and clinical parameters | Boenicke et al. 2017 |
| 5 | Diagnosis and management of a cryptoglandular actinomycotic fistula-in-ano: an update on 7 new cases and a review of the literature | Egal et al. 2018 |
| 6 | Design and psychometric evaluation of the Quality of Life in Patients with Anal Fistula Questionnaire | Ferrer-Márquez et al. 2017 |
| 7 | Analysis and description of disease-specific quality of life in patients with anal fistula | Ferrer-Márquez et al. 2018 |
| 8 | Combined treatment approach to chronic anal fissure with associated anal fistula | Fitz Dowse et al. 2018 |
| 9 | Anorectal conditions: anal fissure and anorectal fistula | Fox et al. 2014 |
| 10 | Anal incontinence and quality of life following operative treatment of simple cryptoglandular fistula-in-ano: a prospective study | Jayarajah et al. 2017 |
| 11 | The type of loose seton for complex anal fistula is essential to improve perianal comfort and quality of life | Kristo et al. 2016 |
| 12 | Fistulotomy and primary sphincteroplasty for anal fistula: long-term data on continence and patient satisfaction | Litta et al. 2019 |
| 13 | Quality of life of patients after surgical treatment of anal fistula; the role of anal manometry | Mylonakis et al. 2001 |
| 14 | Quality of life with anal fistula | Owen et al. 2016 |
| 15 | Quality of life following fistulotomy - short term follow-up | Owen et al. 2017 |
| 16 | Cryptoglandular anal fistula | Parades et al. 2010 |
| 17 | Quality of life following surgery for recurrent fistula-in-ano | Seneviratne et al. 2009 |
| 18 | Ligation of the intersphincteric fistula tract (LIFT) to treat anal fistula: early results from a prospective observational study | Sileri et al. 2011 |
| 19 | Long-term outcomes and quality of life following ligation of the intersphincteric fistula tract for high transsphincteric fistulas | Sun et al. 2019 |
| 20 | Long-term follow-up after surgery for simple and complex cryptoglandular fistulas: faecal incontinence and impact on quality of life | Visscher et al. 2015 |

## Table S6 Responses from patients describing abscess (among the most salient symptoms with a combination of a high frequency and a high disturbance rating)

| *“Then the summer of 2019,* ***I noticed on my right side I had a pretty big cyst or abscess*** *that formed in the same exact spot as my left side. But my right side was like a* ***big lump that had formed and this one was painful.*** *It kind of formed to a golf ball sized lump kind of on the crease of my butt cheek and my leg. That is actually what made me go in to see my gynecologist again and say look, there’s something else going on.”* (Patient 6, female, 34 years old) |
| --- |
| *“When they did the* ***MRI****, it* ***came back that I had a five-inch abscess*** *going down to my tailbone.”* (Patient 7, female, 43 years old) |
| *“****You can feel it.*** *I push on mine or squeeze it in the shower or whatever. You could feel it. First of all, it hurts.* ***It hurts.*** *For me, it's a tolerable hurt because it's not as bad as it was when I went to the emergency room.”* (Patient 7, female, 43 years old) |
| *“You start thinking* ***maybe, oh, is this a cancerous bump*** *or… that kind of thing.”* (Patient 11, male, 64 years old) |

## Table S7 Responses from patients describing odour (among the most salient symptoms with a combination of a high frequency and a high disturbance rating)

| *“It was a* ***stinky, foul*** *odor, like something died.”* (Patient 1, male, 47 years old) |
| --- |
| *“And then the smell. And there was an* ***awful, awful, wretched smell****, and I couldn’t deal with it.”* (Patient 12, female, 41 years old) |
| *“I would* ***rather have the pain*** *than the smell almost.”* (Patient 13, male, 49 years old) |
| *“That was just* ***nasty****. That's going to be a 10. At its worst it was* ***disgusting****. Because that smell was* ***horrible****.”* (Patient 16, female, 32 years old) |
| *“****What really triggered the doctor’s visit*** *was the increase of how it was* ***feeling to go to the restroom*** *and* ***then the smell*** *that it would bring on after a period of time. I thought something might be wrong.”* (Patient 2, female, 57 years old) |
| *“Well, at* ***first it was during bowel movements*** *and* ***then it started to happen…like any time I would go to sit down****, I could definitely smell it. And I was like, something is wrong. Something is really wrong and* ***that’s when I went in****.”* (Patient 12, female, 41 years old) |
| *“It* ***really freaked me out****, that smell. It* ***smelled rotten****, like something was really, really wrong…because I know I didn’t eat food that was bad. I just, I freaked out because of the blood and the smell so* ***I went to go see a doctor****.”* (Patient 14, female, 51 years old) |
| *“I think the seton, when they put the* ***seton*** *in, it just* ***caused the drainage****. Some* ***kind of mucus, smelly…was coming out*** *for about two months when he put the seton in. Like a drainage that smelled. Smelled up the whole bathroom.* ***Stunk so bad****.”* (Patient 1, male, 47 years old) |
| *“There was a* ***foul smell and also white pus draining*** *out of it.”* (Patient 8, male, 54 years old) |
| *“There was a* ***foul smell*** *coming… For me on a personal level, like I said, as somebody who considers themselves and takes care of themselves and practices really good hygiene… It kind of struck me as like, what the heck? I think* ***it was pretty embarrassing****. I was actually* ***to the point where it was like I didn’t feel like sleeping in the same bed with my wife****...”* (Patient 8, male, 54 years old) |
| *“I am pretty good at taking care of myself back there so… When I have that* ***odd smell and everything****, it makes you a little* ***self-conscious****… You’re* ***wondering if other people can smell something*** *that you can’t, while you’re out walking around and that kind of thing.”* (Patient 11, male, 64 years old) |
| *“Just* ***with the smell alone*** *and the pain a little bit, too, the sitting uncomfortableness, I would say that affected quite…that was quite a big impact. I* ***didn’t want to go anywhere****.”* (Patient 15, female, 34 years old) |
| *“Just* ***with my husband, even my kids were calling me stinky****. I know kids don’t really mean harm or anything, but just my husband really…he was concerned about the smell and things like that, too, the cleanliness.”* (Patient 15, female, 34 years old) |

## Table S8 Responses from patients describing pain when having bowel movement (among the most salient symptoms with a combination of a high frequency and a high disturbance rating)

| *“The first time I recognized myself having pain was when I was trying to use the restroom and I experienced some pain in that. That was, as I mentioned, a* ***few months before I was diagnosed****.”* (Patient 2, female, 57 years old) |
| --- |
| *“I had noticed probably you could say the beginning of November, I had started noticing pain when I would go to the bathroom. It just* ***kept getting worse and worse****.”* (Patient 15, female, 34 years old) |
| *“I was being* ***irritated a little bit sometimes when I was pooping****. I was kind of like…there was still* ***swelling*** *around my anus. Something was* ***inflamed****.”* (Patient 1, male, 47 years old) |
| *“How it affects it is when it* ***swells up****. It obviously* ***causes inflammation****, so it swells that area up. Mine is on my left side. It's* ***affecting my sphincter*** *on the left side. How that* ***affects the bowel movement*** *is if it's swollen right there and I'm trying to evacuate the bowel movement, if that muscle is inflamed or full because of the inflammation then it's* ***harder for me to push anything out****. Then it doesn't evacuate fully because you need those muscles to get that out of there…it’s definitely painful…”* (Patient 7, female, 43 years old) |
| *“Going to the bathroom it was painful. I* ***needed to completely change my diet****. I don’t know, I guess my body* ***wasn’t absorbing a lot of fibers, or I was always constipated****, and that was making it worse because every time I would try and go to the bathroom it was just making it worse, and it hurt.”* (Patient 18, male, 44 years old) |
| *“What happened, I started getting pain when I’m having bowel movements. But I, thinking about the constipation, people do constipate. So* ***I thought maybe this comes because my stool is not soft enough****. Okay. And then I started having more pain.”* (Patient 20, female, 69 years old) |
| *“It wasn’t really painful in the beginning before I went to the restroom. But* ***when I was trying to go to the restroom was really where I noticed the pain level****.”* (Patient 2, female, 57 years old) |
| *“The* ***pain is aftershock of using the bathroom****. It was kind of hard for me to even sit still, sometimes on it. Especially, toward the end of the two weeks. That’s why I end up going and seeing a doctor, to see what was going on.”* (Patient 4, female, 48 years old) |
| *“After going to the bathroom. The* ***pain would stay with me for a while****.”* (Patient 4, female, 48 years old) |

## Table S9 Responses from patients describing discomfort (among the most salient impacts with a combination of a high frequency and a high disturbance rating)

| *“The* ***drain****, I noticed a lot of leakage coming out of me. I* ***had to wear a pad. It was uncomfortable****.”* (Patient 2, female, 57 years old) |
| --- |
| *“It was* ***mainly just being uncomfortable during bowel movements****. Maybe just* ***general discomfort****.”* (Patient 3, male, 59 years old) |
| *“****You’ve got a drain coming out****, so it’s kind of* ***awkward to be sitting, or laying****, so that was* ***very uncomfortable****, especially the first week.”* (Patient 12, female, 41 years old) |

## Table S10 Responses from patients describing embarrassment (among the most salient impacts with a combination of a high frequency and a high disturbance rating)

| *“I would have to say with my wife I felt* ***just embarrassed by the whole thing, just the smell****, and I just* ***couldn't really get intimate with her*** *because it bothered me.”* (Patient 9, male, 42 years old) |
| --- |
| *“And then also just like can anybody else smell this?* ***Obviously, I can’t be the only one that smells this. So there’s that shame, like embarrassment factor****…”* (Patient 12, female, 41 years old) |
| *“I was* ***scared to maybe have flatulence****, or something, and somebody smell that and just know that it came from me. It was* ***actually very embarrassing****.”* (Patient 14, female, 51 years old) |
| *“It was so* ***embarrassing****. It was like when I would walk,* ***I would move gas and it was uncontrollable****, actually. It wasn’t where I had any control over it, it was just coming out. That’s why pretty much* ***I tried to isolate myself*** *because we don’t know when it’s going to happen where.”* (Patient 19, female, 64 years old) |
| *“The* ***first time where they did the examination*** *where they put their finger in the colon and looked for that little thing, I* ***was very embarrassed****.”* (Patient 18, male, 44 years old) |
| *“****How can you say I’ve got this bump on my butt and it’s hard for me to use the bathroom****. It smells bad. When I walk there’s flatulence. When I’m sitting down there’s flatulence.* ***How can you say that to someone, even family?*** *It’s hard to…I didn’t speak to anyone about it until after I came from the doctor. Yeah, to me,* ***it was that embarrassing****.”* (Patient 19, female, 64 years old) |
| *“My* ***mood was affected throughout****. Being concerned and fearful and then having the embarrassment, it can’t help but affect your mood.”* (Patient 2, female, 57 years old) |
| *“It was a* ***smell very bad, so it was very embarrassing*** *to me, to my husband, to stay next to the people. I think everybody can smell this smell, whatever. It was really embarrassing. I stopped going anywhere and I [was] washing myself all the time. I didn’t understand what it is. He said it’s a hole. I found out later on it’s a hole, after I went to see the specialist. But that was really upsetting me. I* ***even got depressed****.”* (Patient 20, female, 69 years old) |

## Table S11 Responses from patients describing worry about disease (among the most salient impacts with a combination of a high frequency and a high disturbance rating)

| *“That would be a 10 simply due to the fact that it’s still sensitive, it’s still a wound that needs to heal, and* ***you worry that it’s going to reverse itself and come back****.”* (Patient 8, male, 54 years old) |
| --- |
| *“So I’m going to knock on wood as we speak, and no, and I…****that’s probably my biggest fear is this ever happening again****. I made diet changes. I drank a ton of water. I’m trying to do the best I can so it’s never to happen again.”* (Patient 12, female, 41 years old) |
| *“Well, it’s affected where if I don’t keep drinking water and if I don‘t keep up with all the fiber, then* ***I’m worried to get that fistula again, that blockage****. So I guess it is still affecting my life.”* (Patient 14, female, 51 years old) |

## Table S12 Responses from patients describing increased preparation/adapt life to maintain hygiene (among the most salient impacts with a combination of a high frequency and a high disturbance rating)

| *“The drain, I* ***noticed a lot of leakage*** *coming out of me. I* ***had to wear a pad****. It was uncomfortable.”* (Patient 2, female, 57 years old) |
| --- |
| *“Well, I have pads with me all the time.* ***Lots of time spent in the bathroom. Lots of baby wipes.*** *Initially, when I was having all the surgeries, I* ***lived with a sitz bath****. A sitz bath, a little enema thing, and you're just* ***cleaning, cleaning, cleaning****.”* (Patient 5, female, 49 years old) |
| *“It's* ***always making sure I'm clean****. If you don't make sure you're clean then, yes, you’re going to smell. It's not going to smell good. Just leakage. It's an extra thing I have to do every single day.”* (Patient 7, female, 43 years old) |

## Table S13 Responses from patients describing negatively impacted social life/isolation (among the most salient impacts with a combination of a high frequency and a high disturbance rating)

| *“Like I said, it* ***limited my social activity****. I didn’t feel like participating in things because you* ***never know when your stomach is going to go off*** *or you’re going to have to run to a restroom.”* (Patient 8, male, 54 years old) |
| --- |
| *“From the time it was going on until the time that it was remedied, I* ***curtailed my social life quite a bit*** *to the point where I* ***wasn’t associating with friends****, going out and doing the things that I felt comfortable doing...You* ***just feel like basically withdrawing****.”* (Patient 8, male, 54 years old) |
| *“Well, with the smell, I* ***didn't really want to be around crowds****. I just kind of felt* ***ashamed of the smell****.”* (Patient 9, male, 42 years old) |
| *“I* ***definitely isolated myself****. But the few close girlfriends that I have, they totally understood, and they would come out and hang with me and stuff like that. So it wasn’t…we just wouldn’t go out.”* (Patient 12, female, 41 years old) |
| *“Oh yeah. there were* ***times where I didn’t want to go because I know that there wasn’t going to be a bathroom nearby*** *and I couldn’t… They would invite me to go horseback riding, hiking, stuff like that. There’s no bathrooms so I opted not to do that.”* (Patient 14, female, 51 years old) |
| *“Like I said, I* ***like to go to restaurants for dinner, with my friends for lunch****. And my husband, too. We like to go out with the kids’ birthdays sometimes. No,* ***I stopped going****. If they have invited me that time, I said, “Oh, I’m very sorry. I’m not feeling good.”* ***Because I’m afraid of the smell****.”* (Patient 20, female, 69 years old) |

## Table S14 Responses from patients describing negatively impacted intimate relationships (among the most salient impacts with a combination of a high frequency and a high disturbance rating)

| *“Even with the symptoms,* ***I think I could have had sex****, but I’m thinking it* ***was more or less psychological*** *in the way of the smell, that foul, putrid… I mean* ***I was stinking up the bedroom*** *and the bathroom.”* (Patient 1, male, 47 years old) |
| --- |
| *“It was impacting a little bit, but* ***more prevalent was wondering what was happening and the fear*** *of not knowing what was going on. So* ***that gets pushed to the side when you’re not sure what’s wrong with yourself****.”* (Patient 2, female, 57 years old) |
| *“****If I get asked out on a date, I don't even feel like meeting anybody*** *to even get to the point of getting intimate because I don't even want to go… You know what I'm saying?”* (Patient 7, female, 43 years old) |
| *“Due to the sensitive area and the pain, it* ***affected my relationship on a romantic scale****. I wasn’t having sex with my wife. I* ***didn’t feel attractive****. I* ***felt more withdrawn****. I wasn’t feeling well, there was pain, it needed to heal, I felt withdrawn, so it really affected me there.”* (Patient 8; male, 54 years old) |
| *“I would have to say with my wife I felt* ***just embarrassed by the whole thing****, just the* ***smell****, and I just* ***couldn't really get intimate with her*** *because it bothered me.”* (Patient 9, male, 42 years old) |

## Figure S1 Preliminary CCF conceptual model based on the literature review


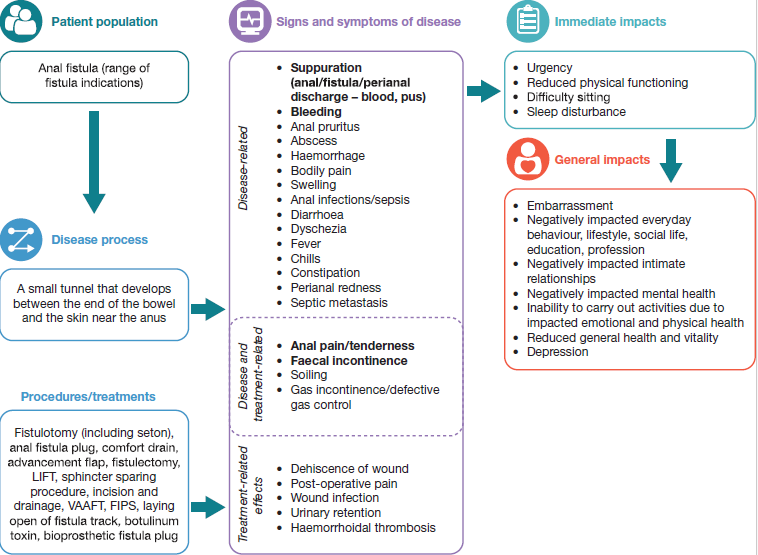
 Bold concepts are ≥50% prevalent as found in the literature. Related concepts were collapsed when constructing the conceptual model. Abbreviations: CCF, complex cryptoglandular fistulas; FIPS, fistulotomy and primary sphincteroplasty; LIFT, ligation of the intersphincteric fistula tract; VAAFT, video-assisted anal fistula treatment.

## **Figure S2** Frequency and mean disturbance rating for the most salient symptoms


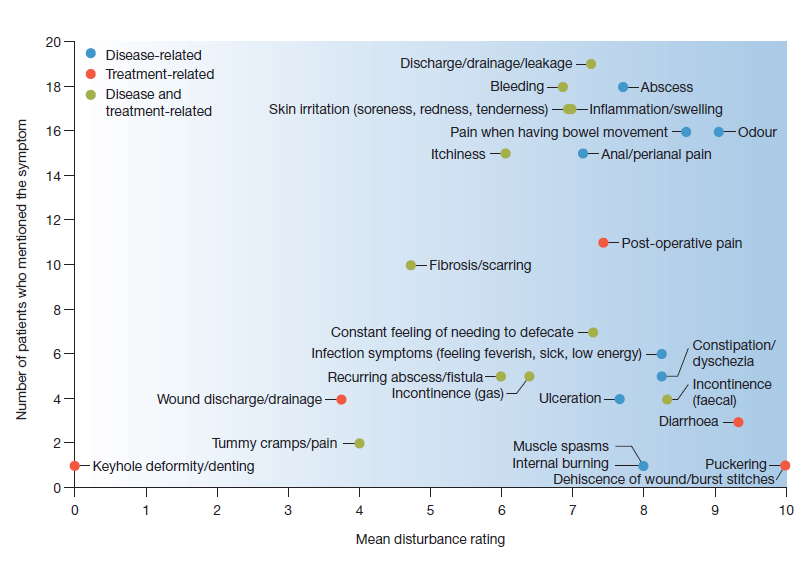
Note: if a range of ratings was provided by the participant, the highest rating was selected.

## Figure S3 Frequency and mean disturbance rating for the most salient impacts


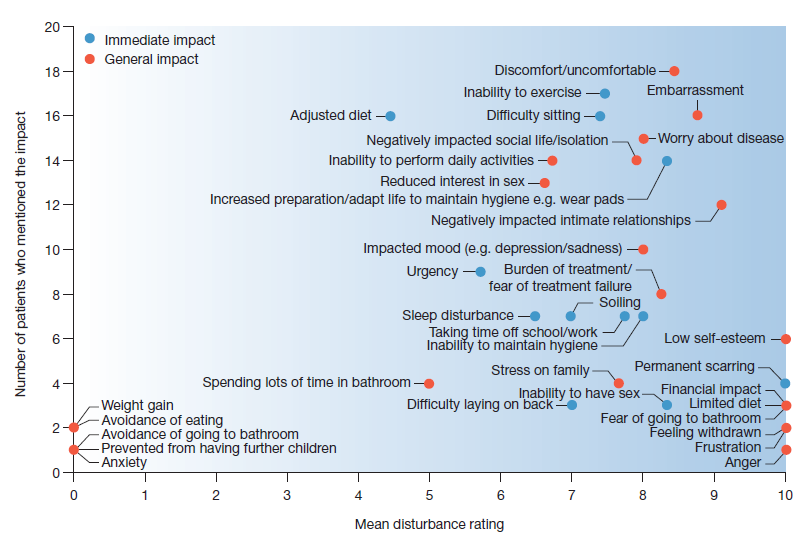


Note: if a range of ratings was provided by the participant, the highest rating was selected.
